# Implications of Physiology-Sensitive Gait Exercise on the Lower Limb Electromyographic Activity of Hemiplegic Post-Stroke Patients: A Feasibility Study in Low Resource Settings

**DOI:** 10.1109/JTEHM.2020.3006181

**Published:** 2020-07-01

**Authors:** Dhaval Solanki, Siddhant Kumar, B. Shubha, Uttama Lahiri

**Affiliations:** Discipline of Electrical EngineeringIIT Gandhinagar 242275 Gandhinagar382355India

**Keywords:** Gait rehabilitation, surface electromyography, treadmill, physiology, stroke

## Abstract

*Background:* Stroke is one of the leading causes of disability with ~80% of post-stroke survivors suffering from gait-related deficits. Conventional gait rehabilitation settings are labor-intensive and need rigorous involvement of clinicians (who use their expertise to decide the dosage of exercise intensity based on patient’s capability). This demands a technology-assisted individualized exercise platform that can offer varying dosage of exercise intensity based on one’s capability. Here, we have used an individualized physiology-sensitive treadmill-assisted gait exercise platform. We wanted to investigate the implications of this platform on one’s lower limb muscle strength and muscle fatigue by analyzing surface Electromyogram (sEMG)-related indices and standard gait-related clinical measures. Methods: We designed a feasibility study involving post-stroke patients (n=9; 44.4(±15)years; post-stroke period=3.1(±3)years) and gave them multiple exposures to this exercise platform. We investigated the Gastrocnemius Lateralis and Tibialis Anterior muscle activation prior to and post multiple exposures to our exercise platform while they walked overground. The feasibility study ended with collecting feedback on the patients’ perception on the implications of having gait exercise using such a platform on their ambulation capability. Results: The results showed that repeated exposures to this gait exercise platform can contribute towards gait rehabilitation of post-stroke patients with rehabilitation outcomes measured in terms of group average improvement of (i)~14% in muscle strength and (ii)marginal (~6%) in functional mobility (as in our case). Conclusion: Such a physiology-sensitive treadmill-assisted gait exercise platform can hold promise towards contributing to post-stroke gait rehabilitation in low-resource settings with the rehabilitation outcomes being measured in terms of sEMG-based observations.

## Graphical abstract

Physiology-sensitive gait exercise platform can offer individualised exercise and it can contribute towards improvement in the muscle strength and functional mobility of post-stroke patients and such a platform can hold promise towards contributing to post-stroke gait rehabilitation in low-resource settings.

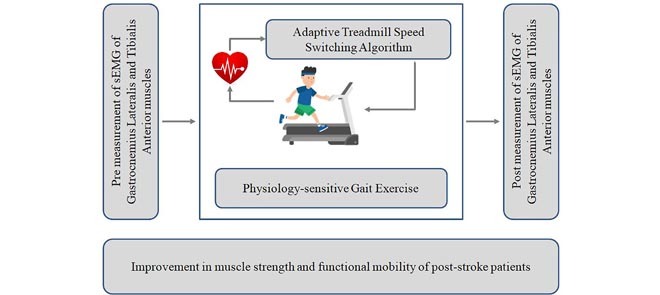

## I. Introduction

Stroke is one of the leading causes of death and disability [1] with prevalence being ~424 per 100,000 people every year in India [2]. Any deconditioned chronic stroke survivor generally exhibits deteriorated ambulation capability with accompanying impaired muscle strength [3]. The deteriorated muscle strength following a stroke often limits the stroke survivor’s capabilities to generate the forces required to initiate and control movements, causing rapid onset of muscle fatigue [3], [4] thereby affecting their mobility [4]. Additionally, literature shows that post-stroke patients often demonstrate almost double the energy expenditure during their ambulation compared to their healthy counterparts [5] with ~2/3^rd^ of the post-stroke survivors exhibiting pathologic gait [6]. Often, deteriorated ambulation capability prevent a stroke survivor from carrying out activities of daily living, earning his/her own livelihood and makes him/her dependent on the caregiver, thereby posing socioeconomic challenges.

To address the deficits related to ambulation, these patients are prescribed gait rehabilitation exercises. The conventional gait rehabilitation therapy often encompasses overground gait exercise along with treadmill-assisted gait exercise. In conventional therapy, while the patient is performing exercise, it is necessary to have continuous involvement of a physiotherapist. This is because the therapist uses his/her expertise to decide the exercise dosage suiting the patient’s capability to do exercise. Though powerful, this can be susceptible to suffer from subjectivity in decision-making and also poses challenges in low-resource settings. This is particularly true in countries like India having limited healthcare resources and high patient-to-doctor ratio [7]. One of the ways to address such challenges can be to use platforms that can enable one clinician to cater to the individualized needs of multiple patients at the same time. This can be achieved by using complementary technology-assisted exercise platforms that need to be equipped with mechanisms that can sense one’s capability to do exercise and accordingly suggest the exercise dosage, thereby saving the clinician’s efforts for other critical activities. One can monitor physiology to sense and quantify one’s capability to do exercise and in turn offer varying dosage of exercise intensity.

Literature shows that monitoring one’s physiological activity can offer estimates of one’s capability to do exercise in terms of energy expenditure during exercise. One’s energy expenditure can be quantified in terms of oxygen consumption during the exercise. However, measurement of oxygen consumption requires a cumbersome and expensive setup that lacks portability and can be unsuitable for use in limited-resource settings. Another alternative to quantify one’s energy expenditure during exercise is measuring heart rate [8]. Researchers have shown that one’s heart rate based proxy measures can be used to estimate energy expenditure during exercise. Additionally, one’s heart rate can be easily monitored using portable, less cumbersome and inexpensive devices [9] than the oxygen monitoring setup and can be easily integrated with any technology-based exercise platform. Given the advantages of incorporating one’s measure of heart rate in the exercise regime, investigators have opted for this alternate technology while offering treadmill-assisted exercises to post-stroke patients. For example, Pohl *et al.* [10] have reported the use of a treadmill-assisted exercise platform in which the exercise intensity was increased after a user’s heart rate have settled down to the respective resting state heart rate. Researchers have gone further to incorporate one’s measure of heart rate in the treadmill-assisted exercise regime. In fact, literature shows that heart rate-controlled treadmill-assisted exercise platforms [11]–[14] with pre-programmed heart rate threshold values have been used to offer varying treadmill speeds thereby increasing exercise intensities for the patients undergoing gait rehabilitation. The rehabilitative outcomes were studied in terms of various spatiotemporal gait parameters. These gait parameters, though powerful indicators of rehabilitation outcomes, such as step length [15], walking speed [15], etc.are mostly explicit in nature. Added to the explicit gait-related measures, one can tap implicit measures, such as lower limb muscle activation. There is a rich history of literature where researchers have studied one’s lower limb muscle activation patterns in connection with treadmill-assisted gait exercise [16]–[18]. But none (to our knowledge) have studied the implications of a heart rate controlled treadmill-assisted gait exercise platform on one’s lower limb muscle activation, particularly for post-stroke gait. Given that post-stroke gait is often characterized by abnormality in lower limb muscle activation pattern [19], reduced muscle strength [20] and fatigue [21], it is crucial to explore the lower limb muscle activation patterns of post-stroke patients undergoing gait exercises with heart rate controlled treadmill-assisted gait exercise platform.

**FIGURE 1.**
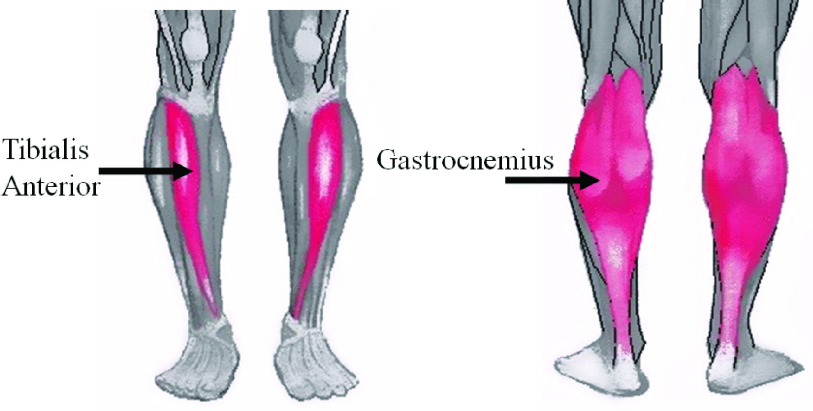
Tibialis Anterior and Gastrocnemius muscles of lower limbs.

One’s muscle activation pattern can be quantified by analyzing the relevant electromyogram (EMG) signals. This is because the EMG signals can be easily accessed non-invasively using portable devices in terms of surface electromyogram (sEMG) and interpreted by technology-assisted platforms. Again, the sEMG signals corresponding to different phases of gait [22] acquired from Gastrocnemius (GL) and Tibialis Anterior (TA) muscles (Fig. 1) [3] can offer signatures of one’s gait pattern [16], [17], [22]. In fact, researchers have studied lower limb muscle activation pattern of GL and TA muscles of post-stroke patients while analyzing their muscle fatigue in terms of mean absolute value and median power frequency of electromyogram [18] during their walk. Again, researchers have studied the root mean square value of the sEMG signals acquired from GL and TA muscles [17], [23] to understand the effect of undergoing treadmill-assisted gait exercise on the increased activation of the lower limb muscles inferring increased muscle strength [17].

Given the importance of studying one’s lower limb muscle (e.g., GL and TA muscles) activation and use of heart rate sensitive treadmill-assisted gait exercise platform for post-stroke patients, in our present research we have investigated the feasibility of use of such physiology-sensitive (i.e., heart rate-sensitive) treadmill-assisted gait exercise to contribute towards post-stroke gait quantified in terms of activation of GL and TA muscles during different gait phases. Again, we hypothesize that exposure to such a physiology-sensitive treadmill-assisted platform will contribute to the improvement in the lower limb muscle strength along with reduction in muscle fatigue thereby leading to reduction in the energy expenditure during gait. The objectives of our research were two-fold, namely to investigate (i) the implication of such a physiology-sensitive treadmill-assisted gait exercise platform on the lower limb muscle strength and muscle fatigue of post-stroke patients through analysis of (a) sEMG-related indices, e.g., mean absolute and root mean square values and (b) standard gait-related clinical measures corroborating the sEMG-based observations and (ii) patients’ perception on the implications of undergoing gait exercise using this platform on their ambulation capability.

This paper is organized as follows: the methodology and apparatus used for the study are described in ‘Methods and Procedures’. The ‘Result and Discussion’ presents the outcomes of the study. Finally, ‘Conclusion’ summarizes the findings, discusses the limitations and future scope.

## II. Methods and Procedures

### A. System Design

Our system comprised of i) Instrumented Shoes, ii) Heart Rate Monitor, iii) Adaptive Treadmill Speed Switching Algorithm and iv) surface Electromyogram Data Acquisition.

#### 1) Instrumented Shoes Module

In our research, we were interested to study the sEMG-based signatures of one’s gait while an individual underwent gait exercise using our Physiology-sensitive treadmill-assisted gait exercise platform. For this, we needed to measure the sEMG signal corresponding to stance and swing phases of one’s gait. These gait phases were identified using Instrumented Shoes (Shoe$_{\mathrm {Sole}}$) having foot switches (made of Force Sensitive Resistors (FSRs)) mounted in the shoe insole at the heel and greater toe positions. The data acquired using the Shoe_Sole_ was stored in a workstation for subsequent offline analysis. This data was used to identify the heel-strike and toe-off events to demarcate the swing and stance phases of a gait cycle [22].

#### 2) Heart Rate Monitor

We recorded one’s Electrocardiogram (ECG) using BIOPAC MP150 (Biopac Inc.) Data Acquisition (DAQ). For this, a three-electrode configuration was used in which Ag/AgCl disposable electrodes were pasted on either side of the neck (aligned with carotid arteries) and fourth intercostal space (towards left parasternal area) [24]. The ECG data was pre-processed and corresponding Heart Rate (HR_WALK_) was computed from the R-R interval [25] and logged into the workstation. The information on HR_WALK_ was (i) fed as an input to the Adaptive Treadmill Speed Switching Algorithm and (ii) used to evaluate Physiological Cost Index (PCI) (Eq. (1)) quantifying one’s energy expenditure [8].

\begin{equation*} PCI=\frac {HR_{WALK}-HR_{REST}}{Walking\mathrm { }~Speed}\tag{1}\end{equation*}

where HR_WALK_ is the HR during walking, HR_REST_ is the HR during the rest period.

#### 3) Adaptive Treadmill Speed Switching Algorithm

The Adaptive Treadmill Speed Switching Algorithm was an in-house built algorithm that was integrated with a motorized treadmill (Fitness World-3100-Motorized Treadmill). The Algorithm was empowered to decide whether to maintain or offer higher challenge by increasing the treadmill speed (based on one’s individualized HR_WALK_) and used a switching criterion. The switching criterion considered one’s age-related maximum HR (HR_MAX_) (Eq. 2) to compute the HR-based exercise threshold zone (HR$_{\mathrm {THRESH\_{}ZONE}}$) belonging to Case 1 and Case 2 (depending on medication [3]) using Eqs. 3 and 4.

\begin{equation*} {\mathrm {HR}}_{\mathrm {MAX}}(beats/minute)=220-Age(in~years)\tag{2}\end{equation*}

Case1: For those not on Beta-Blocker medication.

\begin{align*} \mathrm {0.8\ast }{\mathrm {HR}}_{\mathrm {MAX}}>&{\mathrm {HR}}_{\mathrm {THRESH\_{}ZONE}} \\\ge&0.5\ast {HR}_{MAX}.\tag{3}\end{align*}

Case2: For those on Beta-Blocker medication.

\begin{align*} \mathrm {0.8\ast }\!\!\left ({\mathrm {0.85\ast }{\mathrm {HR}}\!_{\mathrm {MAX\!}} }\right)\!\!>\!\!{\mathrm {HR}}_{\mathrm {\!THRESH\_{}ZONE}}\mathrm {\ge \! 0.5\ast }\!\!\left ({\mathrm {0.85\ast }{\mathrm {HR}}_{\mathrm {MAX}}\! }\right) \\\tag{4}\end{align*}

Depending on one’s HR_WALK_ and bounds of HR$_{\mathrm {THRESH\_{}ZONE}}$, the Algorithm adjusted the treadmill speed. If one’s HR_WALK_ during one trial while using the treadmill was lower than that in the preceding trial, then the Algorithm increased the treadmill speed thereby increasing the exercise intensity. Otherwise, the speed was maintained. The Algorithm terminated the treadmill exercise when one’s HR_WALK_ exceeded the upper bound of HR$_{\mathrm {THRESH\_{}ZONE}}$. If one was able to cope up with increased treadmill speed, our system offered audio feedback e.g., “You are doing great, Keep it up”, otherwise, it delivered “Keep trying, You can do better”.

#### 4) Surface Electromyogram Data Acquisition

In our study, we wanted to investigate the lower limb muscle activation pattern while one performed overground and treadmill-assisted walk. For this, we recorded the sEMG signals from two lower extremity muscles e.g., Gastrocnemius Lateralis (GL) and Tibialis Anterior (TA) [16], [17], [22]. The signals were acquired using adhesive, disposable Ag/AgCl electrodes (3-electrode configuration following SENIAM (Surface EMG for Non-Invasive Assessment of Muscles) standard [26]) and BIOPAC MP150 data acquisition module. The electrodes were placed at the muscle belly of the relevant muscles while avoiding any possible crosstalk with an inter-electrode distance ≤20 mm. A reference electrode was placed on the medial surface of the Tibia. The acquired data was processed to extract time and frequency-domain indices. Specifically, we wanted to study the Mean Absolute Value (MAV), Root Mean Square (RMS), Mean Frequency (MF) and Median Frequency (MDF) to investigate the implications of Physiology-sensitive treadmill-assisted gait exercise on one’s muscle activation and muscle strength [27] along with muscle fatigue [18].

#### 5) Pre-Processing of sEMG Data

The sEMG data was collected wirelessly from the GL and TA muscles at 1000 samples/second via the DAQ and communicated to the workstation. The sEMG signals were filtered using a 2–200 Hz, 5^th^ order butterworth band-pass filter to remove motion artifacts and high-frequency noise [28]. Subsequently, the sEMG signal was processed to remove power line noise using a 50 Hz, 2^nd^ order notch filter followed by smoothening of sEMG signal using a delay adjusted moving average filter (time constant = 50 ms) [29]. The sEMG signal was analyzed to extracts EMG-related indices corresponding to the different gait phases, as identified by the Instrumented Shoes.

#### 6) Extraction of Time-Domain and Frequency-Domain sEMG-Related Indices

We extracted time-domain sEMG-related indices e.g., Mean Absolute Value (MAV) and Root-Mean Square (RMS) [18] from the pre-processed sEMG data using equations (5) and (6), respectively.

\begin{align*} MAV=&\frac {\sum \nolimits _{i=1}^{N} \left |{ x_{i} }\right | }{N} \tag{5}\\ RMS=&\sqrt {\frac {\sum \nolimits _{i=1}^{N} x_{i}^{2} }{N}}\tag{6}\end{align*}

Here, $N$ is the number of samples. The mean frequency (MF) and median frequency (MDF) were calculated [18] using equations (7) and (8), respectively.

\begin{align*} Meanfreq\left ({MF }\right)=&\frac {\int _{0}^{\frac {f_{s}}{2}} {fP\left ({f }\right)df}}{\int _{0}^{\frac {f_{s}}{2}} {P\left ({f }\right)df}} \tag{7}\\ \int _{0}^{f_{med}} P\left ({f }\right)df=&\frac {1}{2}\int _{0}^{\frac {f_{s}}{2}} P\left ({f }\right)df\tag{8}\end{align*}

Here, f_s_ is the sampling frequency, f_med_ is the median frequency and P(f) is the Power Spectral Density (PSD) of the signal computed using a Kaiser window.

### B. Experiment and Methods Participants

In our present work, we have designed a study with post-stroke hemiplegic participants (S_Group henceforth; n = 15, Mean(SD)=48.33(±13.46)years). The recruitment of S_Group participants (who volunteered for the study) was based on a purely convenience sampling. While enrolling, care was taken that all the participants were capable of taking part in a 10 m overground walk test without any external support, could understand the experimenter’s instructions and have not undergone any major surgery in the recent past. Additionally, patients in the acute stage of stroke were not recruited. Out of the 15 post-stroke volunteers, 6 patients discontinued from the study due to personal reasons and their data was not included in the subsequent data analysis. The patients belonged to different ambulation categories [30]. Table 1 shows the participants’ characteristics. These patients were recruited from neighboring hospitals, such as Civil Hospital (Ahmedabad) and C M P College of Physiotherapy (Gandhinagar). The study protocol was reviewed and approved by the institutional ethics committee with the approved proposal number as IEC/2014-15/2/UL/003.

**TABLE 1:** Participant Characteristics; ‘R’ Stands for Right Affected Side and ‘L’ Stands for Left Affected Side

ID	Age (y)	PSP (y)	BMI	Affected Side	AC
P1	37	1.5	29.05	R	LLCW
P2	55	1.5	23.81	R	LLCW
P3	45	2	24.02	R	CW
P4	47	2	23.32	L	LLCW
P5	45	2.5	21.63	R	CW
P6	75	6	27.47	L	MLCW
P7	38	1	29.40	R	LLCW
P8	20	10	20.06	R	CW
P9	38	1	28.93	R	CW

Note: ‘PSP’-Post-stroke period; ‘y’- year; ‘AC’ – Ambulation Category; ‘MLCW’- Most Limited Community Walker (speed<0.6 m/s); ‘LLCW’- Least Limited Community Walker (speed<0.8 m/s); ‘CW’- Community Walker (speed≥0.8 m/s).

#### 1) Experimental Setup

**FIGURE 2.**
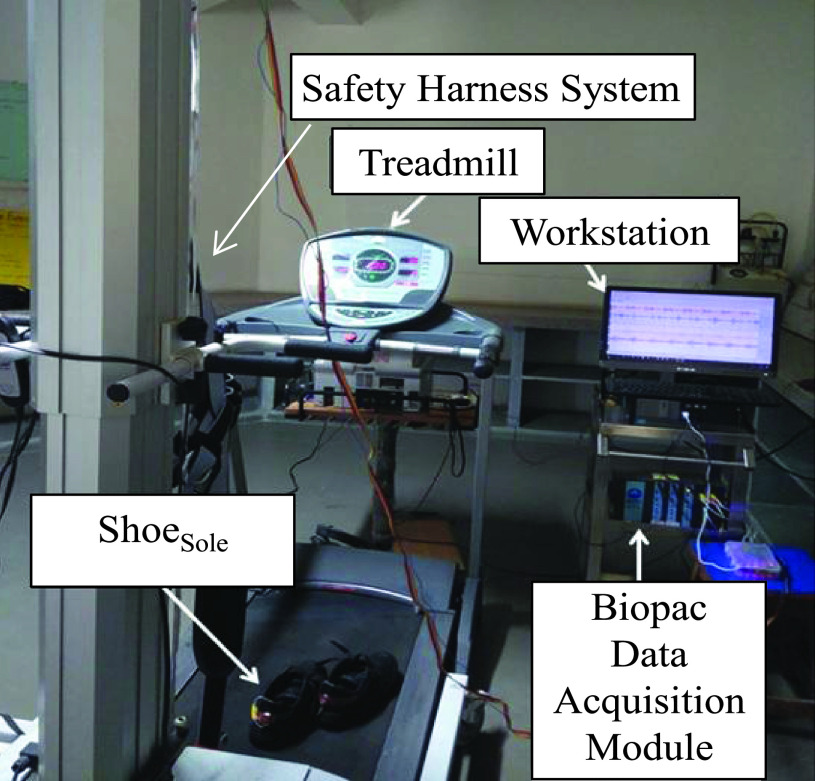
Experimental setup.

The experimental set-up (Fig. 2) included (a) workstation (Intel(R) core(TM) i7-4770 CPU), (b) BIOPAC MP150 DAQ unit to acquire the ECG and sEMG data in wireless mode, (c) treadmill and (d) Instrumented Shoes (Shoe_Sole_).

#### 2) Procedure

In our present research, we designed a feasibility study that exposed post-stroke participants to the Physiology-sensitive Treadmill-assisted gait exercise platform. A commitment of ~30 minutes per session was required from each volunteer. After collecting the participant’s demographic details, the experimenter showed the data collection setup and explained the purpose of the study to the participant. Each participant was informed that he could discontinue from the study at any time in case of inconvenience. The experimenter told the participants that they will be offered 10 sessions (namely first session (Session_FIRST_), intermediate eight sessions and last session (Session_LAST_ *henceforth*) of gait exercise spanning over ~1 month while using our Physiology-sensitive Treadmill-assisted gait exercise platform. Each session comprised of a resting state baseline measurement, a 10m overground walk, a brief warm-up period with the treadmill and treadmill-assisted gait exercise for 10 minutes (incorporating 10 task trials of 1 minute each). The study began after obtaining the signed consent. A physiotherapist in our team examined the medical records (to confirm that any medical condition other than stroke was not responsible for affecting their gait) and conducted clinical evaluation of Motricity (indicative of muscle strength in the lower limbs) [31] and Rivermead Mobility (indicative of functional mobility) [32] Indices prior to Session_FIRST_ and Session_LAST_. The experimenter helped the participant to wear the Shoe_Sole_ and applied electrodes post skin preparation. A harness was used to ensure safety during treadmill-assisted walk. No body-weight support was offered to the post-stroke patients.

Each session began with collection of resting-state ECG and sEMG data as baseline measures. Following this, the participant was asked to walk overground on a 10m long straight pathway using self-selected speed prior to walking on the treadmill. After a brief warm-up period with the treadmill, the participant was exposed to the physiology-sensitive treadmill-assisted gait exercise lasting for 10 minutes. Simultaneously, one’s HR_WALK_ and sEMG-related indices were recorded. Further, at the end of the Session_LAST_, i.e., on the last day of the exposure to the gait exercise platform, the experimenter collected feedback from each post-stroke participant through an informal interview. In this, each participant was asked “Did you realize any alteration in your manner of walk post exposure to our physiology-sensitive gait exercise platform?”. The idea was to hear them on what they think regarding their gait ability based on their daily-living experiences.

### C. Statistical Analysis

Considering the variation in sEMG-related indices across participants, we normalized all the indices on a 0–1 scale (across each session for each participant). While there are maximal and sub-maximal approaches to normalization [33], we used the sub-maximal approach with peak dynamic method of normalization since our participants were post-stroke patients who possessed gait disorders. Our intent was to study the use of sEMG-related indices to understand implications of gait exercise using the physiology-sensitive treadmill-assisted gait exercise platform on one’s gait. For this we carried out comparative analysis between the time-domain (MAV and RMS) and frequency-domain (MF and MDF) sEMG-related indices during one’s overground walk prior to Session_FIRST_ and that prior to Session_LAST_.

Additionally, as a step towards understanding the clinical significance of the changes in sEMG-related indices, we carried out a comparative analysis of the standard gait-related clinical measures, such as Motricity Index quantifying muscle strength and Rivermead Mobility Index quantifying the functional mobility during overground walk prior to Session_FIRST_ and Session_LAST_. Subsequently, we investigated whether the standard gait-related clinical measures corroborated with the sEMG-based observations. Given a limited sample size, we opted for non-parametric statistical test, such as Wilcoxon Signed-rank Test [34].

## III. Results and Discussion

While the participants volunteered in the overground (Walk_OG_ *henceforth*) and physiology-sensitive treadmill-assisted walk (Walk_TREAD_ *henceforth*) exercises, we computed their sEMG-related indices corresponding to stance and swing phases as identified by the Shoe_Sole_. Our aim was to investigate the implication of the physiology-sensitive treadmill-assisted gait exercise platform on one’s lower limb muscle strength and muscle fatigue in terms of time-domain and frequency-domain sEMG-related indices. For this, we carried out analysis of the standard gait-related clinical measures to understand whether these can corroborate the sEMG-based observations. Finally, we wanted to understand one’s perception on the implications of undergoing gait exercise using such a platform on their ambulation capability.

### A. Implication of Physiology-Sensitive Treadmill-Assisted Gait Exercise Platform on sEMG-Related Indices

While the participants took part in the feasibility study, we wanted to investigate the implication of the physiology-sensitive treadmill-assisted gait exercise platform on the muscle strength and muscle fatigue of post-stroke survivors in terms of the time-domain and frequency-domain sEMG-related Indices. We examined the sEMG pattern and analyzed the sEMG-related indices for Walk_OG_ prior to each of Session_FIRST_(*Pre* Walk_OG_) and Session_LAST_(*Post* Walk_OG_).

*Comparative Analysis of Time Domain sEMG-related Indices during Pre*Walk_OG_ and *Post*Walk_OG_

**FIGURE 3.**
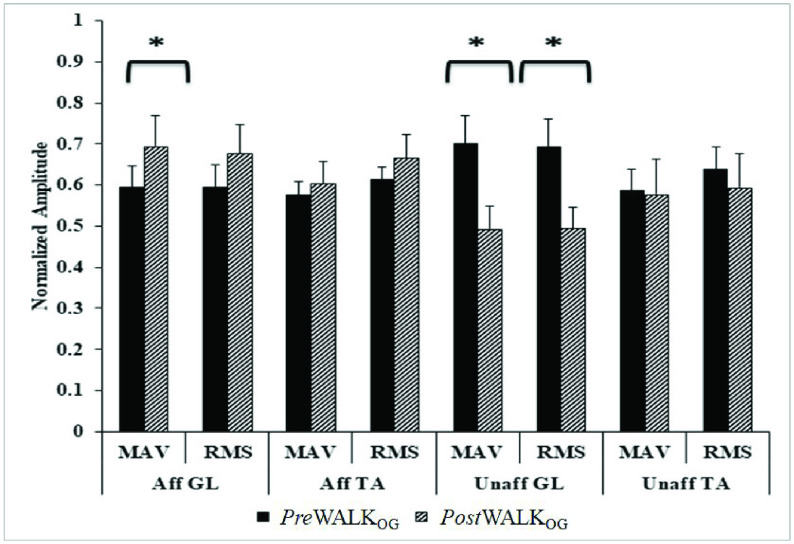
Comparative analysis of sEMG-related time-domain indices during *Pre* Walk_OG_ and *Post* Walk_OG_ in S_Group. Note: Aff (Unaff) GL (TA): Gastrocnemius Lateralis (Tibialis Anterior) for Affected (Unaffected) leg.

We wanted to investigate the sEMG-related time-domain indices, such as MAV and RMS values [17], [18], [23] of the post-stroke patients undergoing the treadmill-assisted gait exercise while they walked overground. The Fig. 3 represents the comparative analysis of sEMG-related time-domain indices for *Pre* Walk_OG_ and *Post* Walk_OG_. It can be seen from Fig. 3 that the MAV and RMS values increased for both the GL and TA muscles from *Pre* Walk_OG_ to *Post* Walk_OG_ on the Affected side of the patients thereby reflecting increased muscle strength of the lower limb muscles of the Affected leg during the overground walk. In contrast, for the Unaffected leg, we see a reduction in the MAV and RMS values for both the GL and TA muscles from *Pre* Walk_OG_ to *Post* Walk_OG_. Such an observation can be attributed to a reduction in compensatory gait strategies [35] being employed by the hemiplegic patients during overground walk. Again, the increase in the sEMG activation in the Unaffected limb can be indicative of reduction in the burden borne by the patients on their Unaffected side during walking thereby contributing to energy-efficient gait. Notwithstanding the fact that such an improved sEMG activation can be at least partly attributed to change in one’s walking speed after multiple exposures to the treadmill-assisted gait exercise platform, yet we can say that such an exposure had implications on one’s gait manifested in terms of improved muscle strength.

We carried out a dependent sample non-parametric statistical test (Wilcoxon signed-rank test) on the MAV and RMS values corresponding to *Pre* Walk_OG_ and *Post* Walk_OG_. For the statistical analysis, we had 16 degrees of freedom and we also analyzed the effect size (r) for the Wilcoxon signed-rank test. For the Affected side, results indicate statistical significance (p-value < 0.05) for MAV of GL, marginal significance for RMS value of both GL and TA muscles. Again, for the Unaffected side, the MAV and RMS of GL were found to be statistically significant (p-value = 0.01, r = 0.6 for both MAV and RMS of GL muscle). In short, we can say that exposures to the physiology-sensitive treadmill-assisted gait exercise platform have contributed to statistical improvement in one’s lower limb muscle strength.

Comparative Analysis of Frequency Domain sEMG-related Indices during PreWalk_OG_ and PostWalk_OG_

**FIGURE 4.**
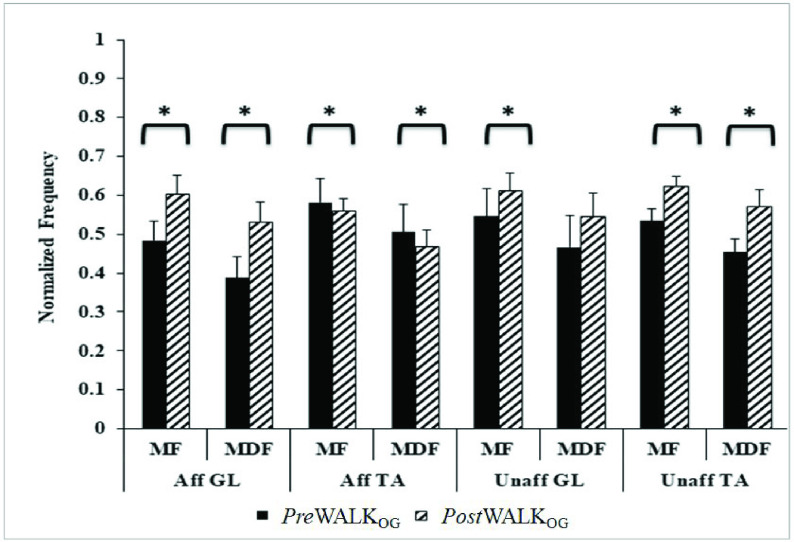
Comparative analysis of sEMG-related frequency-domain indices during *Pre* Walk_OG_ and *Post* Walk_OG_ in S_Group. Note: Aff (Unaff) GL (TA): Gastrocnemius Lateralis (Tibialis Anterior) for Affected (Unaffected) leg.

Added to the time-domain indices, we wanted to explore the frequency-domain sEMG-related indices, such as MF and MDF [18] of the post-stroke patients undergoing the treadmill-assisted gait exercise while they walked overground. The Fig. 4 represents the comparative analysis of sEMG-related frequency-domain indices for *Pre* Walk_OG_ and *Post* Walk_OG_. It can be seen from the Fig. 4 that both the MF and MDF values of the GL muscles increased bilaterally from *Pre* Walk_OG_ to *Post* Walk_OG_. However, for the TA muscle, we observed a decrease in the MF and MDF values for the Affected leg that was not the case for the Unaffected leg. An increase in the MF and MDF values for the Unaffected side might imply a reduction in the muscle fatigue. Such an inference can be bolstered by quantifying the energy expenditure in terms of Physiological Cost Index (PCI) (Eq. (1)). In fact, we found a reduction in the PCI value from *Pre* Walk_OG_ to *Post* Walk_OG_ thereby indicating reduced energy expenditure that in turn might be related to reduced muscle fatigue. A similar inference can be drawn for the GL muscle of the Affected side. In contrast, the variation in the decrease in the MF and MDF values of the TA muscles for the Affected and Unaffected legs might be due to the fact that the hemiplegic patients were making increased usage of their respective TA muscles on the Affected side to achieve better toe clearance while undergoing walk during *Post* Walk_OG_ as compared to that during *Pre* Walk_OG_. This was in line with the observations made by the physiotherapist in our team.

We carried out a dependent sample non-parametric statistical test (Wilcoxon signed-rank test) on the MF and MDF values corresponding to *Pre* Walk_OG_ and *Post* Walk_OG_. The MF and MDF values for GL and TA muscles were found to be statistically different (p-value = 0.02, r = 0.56 for MF and p-value = 0.09, r = 0.39 for MDF of GL muscle; p-value = 0.03, r = 0.51 for MF and p-value = 0.02, r = 0.54 for MDF of TA muscle) for the Unaffected side and (p-value = 0.04, r = 0.49 for MF and p-value = 0.05, r = 0.46 for MDF of GL muscle; p-value = 0.02, r = 0.56 for MF and p-value = 0.02, r = 0.56 for MDF of TA muscle) for the Affected side between the *Pre* Walk_OG_ and *Post* Walk_OG_, except that for MDF for GL in the Unaffected side. To summarize, we can say that exposures to the physiology-sensitive treadmill-assisted gait exercise platform have contributed to statistical reduction in the muscle fatigue.

Morphology of the sEMG Pattern during PreWalk_OG_ and PostWalk_OG_: $A$ Case Study

**FIGURE 5.**
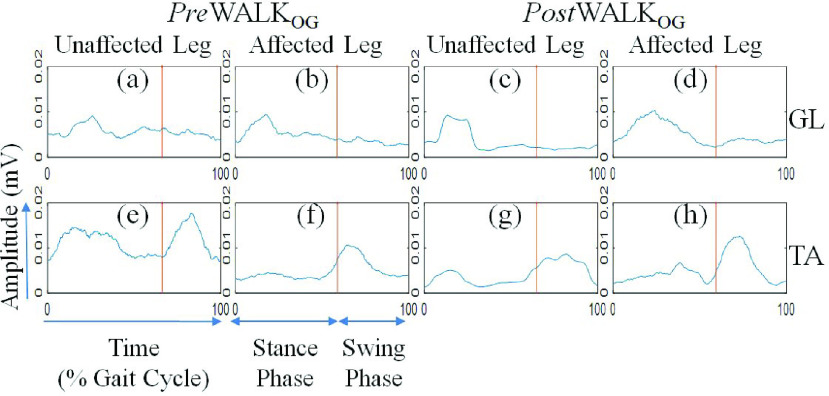
Morphology of sEMG signal from (a) GL muscle (Unaffected) *Pre* Walk_OG_, (b) GL muscle (Affected) *Pre* Walk_OG_, (c) GL muscle (Unaffected) *Post* Walk_OG_, (d) GL muscle (Affected) *Post* Walk_OG_. (e) TA muscle (Unaffected) *Pre* Walk_OG_, (f) TA muscle (Affected) *Pre* Walk_OG_, (g) TA muscle (Unaffected) *Post* Walk_OG_, (h) TA muscle (Affected) *Post* Walk_OG_, Note: GL: Gastrocnemius Lateralis; TA: Tibialis Anterior.

Added to investigating the gait-related indices, we studied the differences in the morphology of the sEMG signal of the post-stroke patients during *Pre* Walk_OG_ and *Post* Walk_OG_ through visual inspection. The Figs. 5(a)-5(h) display the envelope of the average rectified sEMG signal of one of the participants (P1; Table 1) for one gait cycle for the TA and GL muscles of the Affected and Unaffected limbs during *Pre* Walk_OG_ and *Post* Walk_OG_. Overall we could observe that the muscle activation was higher during *Post* Walk_OG_ than that during the *Pre* Walk_OG_ (Figs. 5(a)-5(h)) for both GL and TA muscles in their respective gait phases (i.e., stance and swing phases, respectively) inferring improvement in muscle strength. While considering Figs. 5(a), 5(e), 5(c) and 5(g), we could observe a reduction in the activation of GL and TA muscles during *Post* Walk_OG_ inferring reduction in compensatory gait mechanisms during one’s walk after multiple exposures to our platform. In other words, it might infer that the post-stroke patients increased the usage of their Affected leg during their walk. Again, from Figs 5(b), 5(d), 5(f) and 5(h), we can see that there was an increment in the GL and TA activity in the Affected leg during *Post* Walk_OG_ as compared to that during *Pre* Walk_OG_ inferring improved muscle activity and muscle strength during one’s walk. Also, improved TA muscle activity in the Affected leg from *Pre* Walk_OG_ to *Post* Walk_OG_(Figs. 5(f) and 5(h)) might be indicative of improved toe clearance capability during the swing phase of one’s overground walk.

### B. Analyzing Standard Gait-Related Clinical Measures Corroborating sEMG-Based Observations

While the participants took part in our study, we recorded their standard gait-related clinical measures, such as Rivermead and Motricity Indices [31], [32] during *Pre* Walk_OG_ and *Post* Walk_OG_. We analyzed the clinical assessment measures and checked to see whether these corroborate with our sEMG-based observations on the muscle strength, fatigue and functional mobility of the post-stroke patients. Table 2 shows that there was an overall marginal improvement ($\Delta $%$=\sim 6$%) in the group average Rivermead Mobility Index of the post-stroke participants during *Post* Walk_OG_ compared with that during *Pre* Walk_OG_. This can be used to infer that there was an improvement in the functional mobility that might contribute to promoting their functional independence [32]. While using this finding to corroborate our sEMG-based observation, we can say that this improvement might be related with reduction in muscle fatigue [36] as reflected from the frequency-domain sEMG-related indices in which we found an overall increase ($\Delta $%$=\sim 14$% and 22%) in the MF and MDF, respectively while considering the GL and TA muscles of the Affected limb from *Pre* Walk_OG_ to *Post* Walk_OG_(Fig. 4). Again, as far as the Motricity Index (Table 2) was concerned, we observed a larger improvement ($\Delta $%$=\sim 14$%) from the *Pre* Walk_OG_ to *Post* Walk_OG_. Such an improvement in the Motricity Index might infer improved muscle strength in the participants’ lower limbs [31]. This is in line with our sEMG-based observations in which we could see that the time-domain sEMG-related indices reflected an overall group average improvement ($\Delta $%$=\sim 10.8$% and 11.2%) in the MAV and RMS, respectively (inferring improvement in muscle strength) while considering the GL and TA muscles of the Affected limb from *Pre* Walk_OG_ to *Post* Walk_OG_ (Fig. 3).

**TABLE 2:** Comparative analysis of Rivermead mobility index and Motricity index during Session_FIRST_ and Session_LAST_

Participant ID	PreWalk_OG_	PostWalk_OG_
Rivermead Mobility Index	13.2 (±1.9)	14 (±0.7)
Motricity Index	70.9 (±15)	80.7 (±10.8)

A dependent sample non-parametric statistical Wilcoxon signed-rank test on the clinical scores revealed a significant improvement only in the Motricity index (p-value = 0.02 and r = 0.56), but not for the Rivermead Mobility Index. To summarize, we can say that exposures to the physiology-sensitive treadmill-assisted gait exercise platform have contributed to variations in the standard gait-related clinical measures (through varying degrees) that corroborated with our sEMG-based observations. In other words, the sEMG-related indices can hold promise towards offering complementary measures for monitoring gait rehabilitation outcomes while patients are exposed to such a physiology-sensitive treadmill-assisted gait exercise platform.

### C. Patients’ Feedback: Investigating the Feasibility to Use Our System by Target Population

We wanted to understand the users’ perception on their ambulation capability based on their experiences after undergoing repeated gait exercise using our physiology-sensitive treadmill-assisted gait exercise platform. For this, we recorded post-study feedback from the patients to understand the implications on their ability to walk with improved toe clearance, heel lift and their endurance while walking for longer distances during their daily activities after undergoing gait exercise using our platform. Different participants narrated varying experiences. For example, P1, P2, P4 and P7 mentioned that they felt that they were able to raise their toes sufficiently helping them to easily avoid any small obstacle on their path post exercise. This might infer improved muscle activation and strength related to TA muscle during walking. Also, they reported that they felt using staircase less difficult than before. This might be due to their improved muscle strength related to GL and TA muscles. Additionally, P4 and P7 said that he could walk longer distances with reduced discomfort in his lower limbs. When asked to specify the nature of the discomfort, he said that earlier he used to feel that his legs were becoming heavy after a short walk. However, now he can walk longer distances at a stretch (without taking breaks in between) since he does not feel that his legs are becoming heavy. It can be seen from literature that this feeling of heaviness in the legs might be associated with muscle fatigue [37]. Thus, we can say that repeated exposures to our gait exercise platform have contributed to reduction in muscle fatigue thereby improving endurance to cover longer distances. Similar observations were reported by P3, P5, P8 and P9. For example, P3 and P9 mentioned that they felt more confident (than before) while walking from their respective houses to the clinic on their own, possibly inferring improved endurance. Also, P6 shared positive experience after undergoing the gait exercise using our platform. Specifically, P6 mentioned that he has reduced his dependence on his walking stick to a considerable extent after about 5 exposures to our platform. This might infer improved muscle strength in his lower limbs since he could rely more on his legs to support him during walking instead of using the walking stick. Though his dependence on his walking stick had reduced, yet we observed that he continued carrying his stick while walking. On asking why he continued to bring his walking stick, he told that though he had reduced the usage of his walking stick, yet he had a fear that he might fall. He expressed his desire to have more exposures to our gait exercise platform.

## IV. Conclusion

Neurological disorders, such as stroke can severely affect one’s muscle activity, thereby deteriorating one’s ambulation capability and functional independence. A deconditioned stroke survivor needs to be offered with gait exercise based on his capabilities. In our present research, we have investigated the use of a physiology-sensitive treadmill-assisted gait exercise platform for offering gait exercise to post-stroke patients in an individualized manner. Specifically, the platform was adaptive to one’s individualized energy expenditure estimated from his / her heart rate and in turn offered treadmill-based varying exercise intensities. Our post-stroke patients were offered multiple exposures to this platform while they took part in a feasibility study. Additionally, we acquired the surface Electromyogram (sEMG) data from one’s lower limb muscles that can offer implicit gait-related measures. The idea was to investigate the implication of such a physiology-sensitive treadmill-assisted gait exercise platform on the lower limb muscle strength and muscle fatigue of post-stroke patients through analysis of the sEMG-related indices. Further, we measured standard gait-related clinical measures to corroborate our sEMG-based observations. The feasibility study ended with collecting feedback to understand the patients’ perception on the implications of having gait exercise using such a platform on their ambulation capability. Results of our study showed that repeated exposures to our physiology-sensitive treadmill-assisted gait exercise platform can contribute towards gait rehabilitation of post-stroke patients with rehabilitation outcomes measured in terms of (i) sEMG-based observations and (ii) standard gait-related clinical measures corroborating the sEMG-based findings.

Though the results are promising, yet our study had some limitations. For example, we had a limited sample size and the post-stroke volunteers had heterogeneous characteristics. Also, the number of exposures given to each patient was limited. While our study was designed as a proof-of-concept and not as a full-fledged rehabilitation work, in future, we plan to extend our work by carrying out longitudinal study involving larger participant pool while segregating the post-stroke patients based on their extent of disability. This might help us to understand whether there exists any relation between the dosage of exercise (that is the number of exposures to our gait exercise platform) and the extent of gait-related disorder. Another limitation is that here we offered varying exercise intensities by changing the treadmill speed. It might so happen that one’s neurological or biomechanical factors can prevent one to cope up with the increased treadmill speed. Presently, to account for such a limitation, we have enrolled patients who could walk on level ground without any external support. Another alternative to offering varying exercise intensity can be to increase the elevation of the treadmill belts while maintaining the treadmill speed. Additionally, a limitation of our presently used physiology-sensitive treadmill-assisted gait exercise platform was that it used BIOPAC MP150 module to measure one’s Heart Rate and sEMG wirelessly. This is an expensive device and comes with wearable transmitters along with tabletop mounted receiver adding to the complexity and cumbersomeness of the platform. With an aim to reduce the cumbersome and complex nature of the connectivities on account of the measurement of heart rate and sEMG, we are now working towards interfacing our system to commercially-available, light-weight, wearable watches (offering heart rate information) and light-weight sEMG modules.

Nonetheless, the findings of our feasibility study show that gait exercise with such a physiology-sensitive treadmill-assisted exercise platform can contribute to improvement in one’s muscle strength and reduce muscle fatigue along with ensuring efficient energy expenditure. Again, such a physiology-sensitive treadmill-assisted exercise platform can offer a complementary tool in the hands of a clinician so that one therapist can cater to multiple patients at the same time, thereby alleviating problems of limited healthcare resources. Thus, such a physiology-sensitive treadmill-assisted gait exercise platform can hold promise towards bringing a paradigm shift in the area of post-stroke gait rehabilitation in low-resource settings.
